# Asthma Control and Medication Reliance Among Asthmatics in a General Practice Setting - A Questionnaire Based Study

**DOI:** 10.7759/cureus.25465

**Published:** 2022-05-29

**Authors:** Susan Guyton, Tony Jackson

**Affiliations:** 1 General Practice, Oceania University of Medicine, Wellington, NZL; 2 General Practice, Newlands Medical Centre, Wellington, NZL

**Keywords:** mild intermittent asthma, new zealand, saba reliance questionnaire, short-acting beta agonist, inhaled corticosteroids, asthma control test, mild persistent asthma

## Abstract

The main goal of asthma treatment is to obtain and maintain reasonable control of symptoms with current evidence-based protocols. Asthma is one of the chronic diseases, that, if not well controlled, can lead to the development of serious complications involving not only the respiratory, but the cardiovascular system. In this study, we aimed to determine how well intermittent asthma and mild persistent asthma are controlled among patients aged 15 years and above in a general practice setting using Asthma Control Test (ACT) questionnaire. Furthermore, we used a SABA Reliance Questionnaire (SRQ) to understand whether these patients rely heavily on their prescribed short-acting beta agonist (SABA) medication. This is an audit questionnaire-based study conducted in our primary health care setting in New Zealand from February to May 2021. Forty-three physician-diagnosed intermittent asthma and mild persistent asthma patients aged 15 years and above volunteered to take part in this study. They completed two questionnaires - the ACT to assess asthma control and the SRQ to assess patients’ reliance on SABA reliever inhalers. Overall, 43 patients responded; asthma was well-controlled in 23 patients (53.5%), partially controlled in 15 (34.9%), and uncontrolled in five (11.6%). The total of patients who showed a high reliance on SABA was 31 (72.1%). When comparing SABA reliance with asthma control, 23 (53.5%) patients reported well-controlled asthma; however, 13 (56.5%) of these patients showed a high reliance on SABA. Furthermore, 15 (34.9%) of the patients had partially controlled asthma, and 14 (93.3%) of them also relied highly on their SABA inhalers. We concluded that intermittent asthma and mild persistent asthma remains inadequately controlled in general practice, with the majority of patients showing a high reliance on SABA.

## Introduction

Asthma is defined as a chronic illness of the lungs characterized by reversible inflammation and bronchiolar narrowing resulting in edema and secretion of mucus. These events lead to acute bronchoconstriction and airflow obstruction resulting in alveolar hypoventilation. Symptoms can be any combination of cough, wheeze, shortness of breath, and chest tightness. Asthma is one of the major non-communicable diseases (NCD) globally, with its prevalence still growing in some developing countries [1,2]. In recent years, deaths resulting from asthma have dropped dramatically. However, national and international research continues to reveal inadequate asthma control in a majority of patients. For example, asthma affected an estimated 262 million people in 2019 and caused 461000 deaths worldwide [2,3].

Internationally, the prevalence of asthma in New Zealand is high, with 13% of children and 12% of adults on asthma medication [4]. In 2018, the Health Quality and Safety Commission of New Zealand reported that 14% of asthmatics had a second hospital readmission within three months, and 40% were not regularly prescribed an Inhaled Corticosteroid (ICS) in the year following their admission. The reports on asthma mortality from 2010 to 2015 were highest for Māori and Pacific people than for those of Other ethnicity, and socioeconomic differences in mortality were observed [5].

Asthma severity is classified into four categories. These are intermittent asthma, mild persistent asthma, moderate persistent asthma, and severe persistent asthma. The characteristics of intermittent asthma symptoms are wheezing and coughing no more than two days a week; night-time flare-ups occur twice a month at most, with normal lung function test of FEV1 being 80% or more. If symptoms appear more often than two days a week or two nights per month, on average, this becomes persistent asthma [1].

In this study, we focus on asthmatic patients with intermittent asthma and mild persistent asthma. The Global Initiative for Asthma (GINA) expert panel had initially recommended short-acting beta agonist (SABA) monotherapy as required for the management of mild intermittent asthma and SABA and low dose ICS for mild persistent asthma. In 2019, GINA guidelines introduced ICS in addition to SABA for asthma management as an early effective anti-inflammatory treatment in even in the mildest presentation of the disease for best asthma control. This is a more effective way of managing the disease than waiting until symptoms have been present for several years before targeting the underlying cause of airway inflammation. Patients' poor understanding of ICS use may lead to poor adherence as they perceive SABA alone to provide quick relief from exacerbation [6,7].

Research shows that most patients increase their use of SABA when symptoms worsen for quick symptoms relief and are less likely to increase the use of ICS. The patients who have self-managed their asthma by using SABA for many years and found quick symptom relief may continue to believe that this is the most effective way to manage their symptoms. This is especially if there is a poor patient-physician correspondence that aims to regularly and adequately review the asthma health outcomes [6-8].

High reliance on SABA may give patients the perception that their asthma is "well-controlled" due to using their reliever immensely and underusing the ICS [7]. Consequently, as per GINA 2019 guidelines, it is best to initiate therapy with anti-inflammatory treatment earlier in the management to improve lung function and optimally control asthma. If patients understand how this therapy works, they might adhere to the recommended approach, which is a combination of SABA and ICS [9].

The control of asthma is typically assessed by reviewing symptoms and measuring airﬂow obstruction using spirometry or peak ﬂow. Recently, validated questionnaires have played an essential role in the assessment of asthma control. The Asthma Control Test (ACT) is a questionnaire developed as an easy method for patients and clinicians to assess symptoms, the use of rescue medication, and impact on activities [10,11]. The ACT has been validated and recognized by many as an effective, patient-friendly tool to assess asthma control with much more convenience and adaptability. This test will be used in this study to evaluate asthma control.

Furthermore, SABA reliance will be assessed using the SABA Reliance Questionnaire (SRQ) [12]. The SRQ identifies patients who believe that their asthma is best managed by SABA alone. It, therefore, assists clinicians in identifying patients who would benefit from having their asthma medication reviewed. Our hypothesis is that the majority of the patients will overestimate how well their asthma is controlled and that these patients may show a high reliance on their SABA medication.

## Materials and methods

Design and setting

An observational, questionnaire-based study was carried out from February to May 2021 in a single general practitioner setting in Wellington, New Zealand. A single general practice database was searched for intermittent asthma and mild persistent asthmatic patients using Salbutamol/SABA as a sole treatment for asthma management. The database had approximately 100 patients diagnosed with mild asthma.

Study participants: Participants in this study were patients aged 15 years or above diagnosed with mild asthma and whose therapy was SABA monotherapy.

Inclusion and exclusion criteria

All individuals aged >15 with an established diagnosis of mild persistent asthma from doctor's records of pulmonary function tests and SABA reversibility of effect, and patients who are currently on SABA prescription alone were included in the study. Patients that had a viral infection with wheeze, and who had moved to another medical practice during the time that the study was being conducted were excluded from the study. Patients under the age of 15 were invited to participate in asthma-related nationwide research during this study.

Data collection and variables

We identified 90 patients, and appointment letters with questionnaires were sent, informing them of the practice audit and a need for their asthma medication review with their doctor. They were requested to complete two questionnaires and return them in a provided self-addressed envelope. They were offered a free appointment with their general practitioner to discuss the current asthma management recommendations. The appointment letter was followed up by a phone call for those patients who had not made an appointment within a fortnight of the letter being sent, and we had forty-three patients respond.

We collected data on age, gender, smoking status, and years on SABA, asthma control, and reliance on SABA. Patients' level of asthma control was measured using the ACT to ascertain symptoms over four weeks. Patients were requested to rate their symptoms using daily activity limitations, their shortness of breath, night-time awakening, and how often they use rescue medications. Most importantly, the participants were asked to rate their asthma control. Each questionnaire with an ACT equal to or less than 19 was considered uncontrolled, scores of 20-24 were considered partially controlled, and scores of 25 points were considered completely controlled.

The level of reliance on SABA was ascertained using SRQ, which is a validated five-item questionnaire used to assess patients' perceptions that could lead them to become over-reliant on SABAs. The assessment of SABA reliance is established by patients strongly agreeing (a score of 5) to strongly disagreeing (a score of 1) with the following statements: i) using my reliever is the best way to stay on top of my asthma; ii) I do not worry about asthma if I have my reliever around; iii) I prefer to rely on my reliever than my preventer; iv) the benefits of using my reliever outweigh any risks, and v) my reliever is the only asthma treatment I can rely on. Scores out of 25, with reliance scores: 5-10 is regarded as low, 11-19 as medium, and 20-25 as high reliance.

Data analysis

Microsoft Excel (Microsoft, Redmond, Washington) was utilized, categorical variables were outlined as frequencies and percentages, and a histogram was generated to present the relationships of variables. 

Ethics approval

The Institutional Review Board, Oceania University of Medicine, approved this research. Permission to access records for the study was obtained from the medical center management prior to data collection. Patients were given information, and their participation was voluntary. 

## Results

The response rate was 48%, with 43 patients included in this study. There were 25 females (58.3%) and 18 males (41.7%). The mean age was 43.7 ± 14.6 years (range: 17-74 years). The mean duration of years on SABA was 12.7, with an interquartile range (IQR) of 7 and 18.5 years. In addition, five (11.6%) reported a history of smoking, and among current smokers, only three (7%) of the sample and 81.4% were non-smokers. Asthma was well controlled in 23 patients (53.5%), partially controlled in 15 (34.9%), and uncontrolled in five (11.6%). High SABA reliance was seen in 31 (72.1%) of participants; seven (16.3%) were medium reliance, and five (11.6%) showed low reliance. Table 1 below outlines the sociodemographic characteristics of the participants.

**Table 1:** Sociodemographic characteristics of the participants SABA - short-acting beta agonist

Variables	Value	Percentage
Gender		
Male	18	41.7%
Female	25	58.3%
Age: mean ± SD, years	43.7+/-14.6	-
Age group		
Under 30	8	18.6%
31-45	20	46.5%
Over 45	15	34.9%
Smoking status		
Current smoker	3	7.0%
Ex-smoker	5	11.6%
Non-smoker	35	81.4%
Years on SABA		
1-10	18	41.9%
11-20	19	44.2%
21-30	6	14%
Asthma control (ACT)		
Well-controlled	23	53.5%
Partially controlled	15	34.9%
Uncontrolled	5	11.6%
Reliance on SABA (SRQ)		
Low reliance	5	11.6%
Medium reliance	7	16.3%
High reliance	31	72.1%

**Figure 1:**
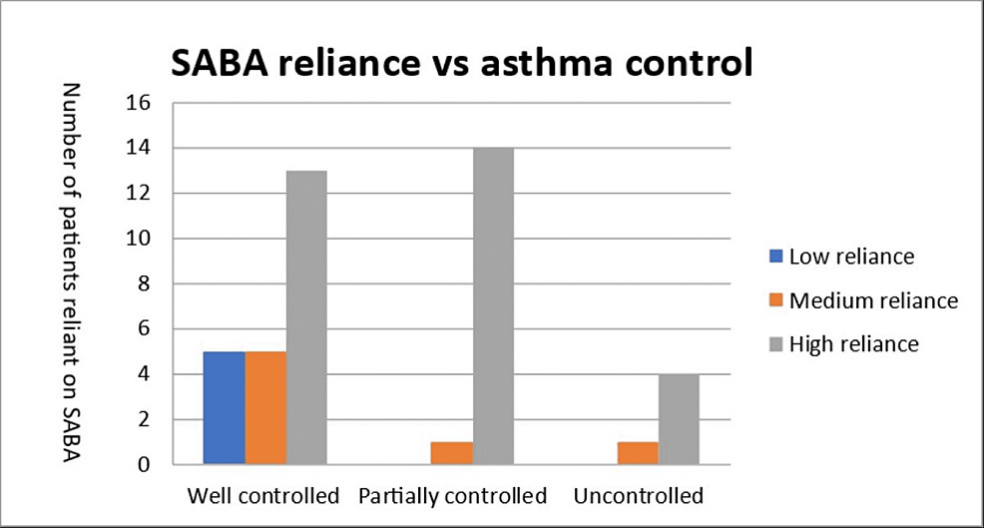
SABA reliance vs. asthma control SABA - short-acting beta agonist

The total of patients who are highly reliant on SABA is 31 (72.1%). When comparing SABA reliance with asthma control, 23 (53.5%) patients reported well-controlled asthma; however, 13 (56.5%) of these patients showed a high reliance on SABA. Furthermore, 15 (34.9%) of the patients had partially controlled asthma, and 14 (93.3%) of them also relied highly on their SABA inhalers. There were five (11.6%) patients in the uncontrolled asthma group, and four (80%) of the patients show a heavy reliance on SABA (Figure 1).

## Discussion

As far as we are aware, this study is the first to look at asthma control in relation to SABAs reliance among asthmatics. It provides a unique "snapshot" of patients with intermittent asthma and mild persistent asthma using SABA relievers. We looked at asthma control in relation to age, gender, smoking status, and years on SABA. 

Whether age affects asthma control, we observed the younger age group (under 30) having more challenges with control. This has also been shown in a Brazilian study where the negative impact of asthma in younger age groups was found [13]. Young people have poor control of asthma due to reduced compliance with treatment [14]. In contrast, a Chinese study aimed at evaluating treatment adherence and causes of non-adherence showed that those in the 30-39 year age group had the worst treatment adherence (27.3%) [15]. Furthermore, other studies have concluded that elderly asthmatic patients have worse short and long-term asthma control than the younger adult population due to aging and preexisting comorbidities [16,17].

Our study found no effect of gender on asthma control. A previous study by Dursum and colleagues compared asthma control in males and females and found no significant statistical difference based on ACT scores and clinical characteristics [18].

When looking at the smoking status and asthma control, we found that non-smokers tend to have better asthma control than smokers. This agrees with other studies showing that smokers with asthma have poorer control of their condition than non-smokers, and mortality is more significant among heavy smokers than non-smokers [19,20].

Patients who have been on SABA for longer, 11 to 20 years, appear to have their asthma well-controlled. There may be a better awareness of symptoms over the years and managing symptoms with SABA. However, 22% of those who have been on SABA for a shorter period (1-10 years) show poor control of the disease. This may be due to the patients being of a younger age group and some recently diagnosed. A qualitative study examined the reasons for SABA over-reliance in young adults with asthma and found that these young patients rely heavily on SABA, including wanting a quick fix and their illness causing them significant frustration [21].

This study found good asthma control, with 53.5% of patients reporting well-controlled asthma as per ACT scores. Nonetheless, the SRQ scores revealed that 72.1% of patients highly rely on their SABA medication even though more than half (56.5%) of them reported well-controlled asthma. It appears that they have their asthma symptoms under control on SABA alone when in actual fact, they are using it a lot more than ICS medication. The use of SABA alone provides quick relaxation of airway smooth muscle, but the addition of ICS inhibits chronic inflammation of the airway and tissue remodeling. The hypothesis of perceived asthma control fits our hypothesis that the majority will overestimate how well their asthma is controlled due to the overuse of SABA without concomitant use of maintenance ICS therapy. It is, therefore, vital that patients and clinicians work together to achieve optimal drug therapy for patients with mild asthma, as supported by current research [22-24].

Similar observations were made by Laforest et al. in a community pharmacy-based survey in France [25]. In their study, patients consulted their GPs monthly, whereby they commonly reported daily shortness of breath (30%), everyday use of SABA (29%), and weekly night-time symptoms (32%). Interestingly, the majority considered their disease completely or well-controlled without the addition of ICS in their treatment (76%) [21]. Another study by Nwaru et al. also investigated patients overusing of SABA and its effect on controlling asthma and found that 30% overused these reliever medications [26]. These researches were conducted in primary care settings, and they demonstrate how the real-life condition of patients has not reached the desired optimal control.

The risk of asthma exacerbations must be addressed in general practitioner offices, and education offered to such patients. When observing the asthma status, a large proportion of participants reported symptoms consistent with partially or controlled asthma on SABA monotherapy. The current recommendations acknowledge evidence-based research that suggests that SABAs overuse in the absence of concomitant controller medications is unsafe as it fails to address the underlying airway inflammatory process which leads to chronic inflammation and detrimental airway narrowing by reducing infiltration of eosinophils in the airways [27-29]. Healthcare professionals in asthma care must understand the perceptions and habits of people with intermittent asthma and mild persistent asthma and use the opportunities to identify at risk of poorly controlled disease.

The strengths of our study are that it highlights SABA's reliance on the perception of well-controlled asthma and, therefore, addresses the need to address poor control of mild and mild persistent intermittent asthma in accordance with the latest guidelines. The limitation of our study is a poor response rate. This might be improved in further studies by attempting to collect data from multiple locations because such an approach would both minimize institutional artifacts and allow for a larger overall sample size. Secondly, we did not assess asthma control using pulmonary function tests. The lack of detailed physical evaluation data is partly a limitation of the used questionnaire format even though the ACT has been validated and correlates with the asthma control level. Thirdly, the addition of ethnicity, education level, and comorbidities of the participants could have added more helpful information to the results. Finally, the study does not have competing interests.

## Conclusions

According to our study, symptoms of mild asthma and mild persistent asthma are inadequately controlled in our practice. This is a likely scenario in other parts of New Zealand and worldwide. The poor asthma control in relation to high reliance on SABA confirmed our hypothesis. This fits with existing literature reported in other parts of the world. Although tools and clinical guidelines are widely available and change is happening, there is still a gap between current guidelines and daily practice. Clinicians need to evaluate asthma patients frequently to ensure that evidence based practice in adhered to. Detailed studies of larger sample sizes are required to contradict or validate our findings. 

## Appendices

**Table 2:** Asthma Control Test

	1	2	3	4	5
In the past 4 weeks, how often did your asthma prevent you from getting as much done at work, school or at home?	All of the time	Most of the time	Some of the time	A little of the time	Not at all
During the past 4 weeks, how often have you had shortness of breath?	More than once a day	Once a day	3 to 6 times a week	Once or twice a week	Not at all
During the past 4 weeks, how often did your asthma symptoms (wheezing, coughing, shortness of breath, chest tightness or pain) wake you up at night or earlier than usual in the morning?	4 or more times a week	2 to 3 nights a week	1 night a week	Less than 1 night a week	Not at all
During the past 4 weeks, how often have you used your blue puffer or reliever medication (such as Ventolin, Salbutamol or Terbutaline)?	3 or more times a day	1 or 2 times per day	2 or 3 times per week	Once a week or less	Not at all
How would you rate your asthma control during the past 4 weeks?	Not controlled	Poorly controlled	Somewhat controlled	Well controlled	Completely controlled

**Table 3:** SABA Reliance Questionnaire

	strongly disagree	disagree	uncertain	agree	strongly agree
1.Using my reliever to treat symptoms is the best way to keep on top of my asthma	1	2	3	4	5
2. I don't worry about asthma when I have my reliever around	1	2	3	4	5
3. My reliever is the only asthma treatment I can really rely on	1	2	3	4	5
4. The benefits of using my reliever inhaler massively outweigh any risks	1	2	3	4	5
5. I prefer to rely on my reliever than my preventer inhaler	1	2	3	4	5
